# Protection Effect of Kallistatin on Carbon Tetrachloride-Induced Liver Fibrosis in Rats via Antioxidative Stress

**DOI:** 10.1371/journal.pone.0088498

**Published:** 2014-02-18

**Authors:** Xiaoping Huang, Xiao Wang, Yinghui Lv, Luli Xu, Junsheng Lin, Yong Diao

**Affiliations:** Institute of Molecular Medicine, Huaqiao University, Quanzhou, China; IIT Research Institute, United States of America

## Abstract

Prolonged inflammation and oxidative stress are emerging as key causes of pathological wound healing and the development of liver fibrosis. We have investigated the effects of recombinant human kallistatin, produced in *Pichia. pastoris*, on preventing carbon tetrachloride (CCl_4_)-induced liver fibrosis in rats. Daily administration of kallistatin prevented development of CCl_4_-induced liver fibrosis, which was evidenced by histological study. In all kallistatin treated rats, activation of hepatic stellate cells (HSC) as assessed by s-smooth muscle actin staining was attenuated, TGF- β1 expression was inhibited, class I serum biomarkers associated with the process of fibrogenesis, such as hyaluronic acid, laminin, and procollagen III, were lowered, compared with that in the model control group. Furthermore, residual hepatic functional reserve was improved by kallistatin treatment. CCl_4_ induced elevation of malondialdehyde level and reduced superoxide dismutase activity in the liver, while kallistatin reduced these oxidative parameters. We also investigated the effects of kallistatin on rat primary HSC and LX-2, the human HSC cell line. Kallistatin scavenged H_2_O_2_-induced ROS in the LX-2 cells, and suppressed the activation of primary HSC. These results suggest recombinant human kallistatin might be a promising drug candidate for therapeutic intervention of liver fibrosis.

## Introduction

Advanced liver fibrosis resulting in cirrhosis, liver failure, portal hypertension and hepatocellular carcinoma (HCC) is one of the major causes of morbidity and mortality worldwide. The only curative treatment for liver cirrhosis is transplantation nowadays. But its wide application is restrained by the limitation of the donor organ availability and the recipient conditions. Research on molecular and cellular pathogenesis of hepatic fibrosis might contribute to provide a complementary approach of liver transplantation. The long-held dogma that liver fibrosis is irreversible and relentlessly progressive has been challenged by the increasing evidences that liver fibrosis is a highly dynamic process [1]. Progress in elucidation of the cellular and molecular mechanisms of hepatic fibrosis has brought us to a juncture where translation of these discoveries into treatments is nearing reality. Numerous clinical and experimental observations have demonstrated that prolonged inflammation and oxidative stress may cause pathological wound healing and the development of liver fibrosis [2]. Therefore, selectively target the detrimental effects of oxidant stress could be a strategy for antifibrotic therapy in the future.

Recent research has shown that kallistatin, a kind of plasma protein that belongs to the serine protease inhibitor family, is closely involved in cellular adaptation to oxidative stress, and the anti-inflammatory response. Kallistatin levels are reduced in the kidney and blood vessels under oxidative stress conditions; depletion of endogenous kallistatin with anti-kallistatin antibody exacerbates renal and cardiovascular oxidative stress and subsequence inflammation [3].

Adenovirus-mediated human SERPINA4 gene (coding kallistatin) delivery reduced the oxidative stress, and prevented salt-induced kidney injury, inflammation and fibrosis by inhibiting the expression levels of reactive oxygen species (ROS)-induced proinflammatory cytokines and transforming growth factor-beta1 (TGF-β1) [4]. Adenovirus-mediated human kallistatin gene delivery also suppressed arthritis by inhibiting inflammation in a rat model of arthritis [5]. Kallistatin gene transfer into rat hearts improved cardiac function and reduced ventricular remodeling, oxidative stress, cardiomyocyte apoptosis, and inflammatory cell accumulation after acute myocardial ischemia/reperfusion and chronic heart failure [6]–[8]. Collectively, these findings indicated that kallistatin may play an important role in the protection against oxidative stress-induced inflammatory responses and organ damage. In our previous study, human kallistatin gene therapy alleviated the carbon tetrachloride (CCl_4_)-induced oxidative stress and inflammatory response, and reduced the liver damage in a mouse model [9]. However, fundamental difficulties remain associated with expression regulation of the transferred gene and viral vector-based immunogenicity.

Functional recombinant human kallistatin has been produced in our laboratory using a yeast expression system [10]. To determine whether the recombinant human kallistatin is beneficial in experimental liver fibrosis, rats were treated with the recombinant human kallistatin. It has been evidenced that the recombinant human kallistatin exhibited hepatoprotective and antifibrotic effects in CCl_4_-induced liver fibrosis by daily intraperitoneal injection.

## Materials and Methods

### Reagents

Recombinant human kallistatin was expressed in *Pichia pastoris* strain GS115 and purified with a series of chromatographic steps, mainly Phenyl Superose and Heparin Sepharose FF chromatography. The rabbit anti-rat TGF-β1 polyclonal antibodies and rabbit anti-rat α-smooth muscle actin (α-SMA) monoclonal antibody were obtained from Biotechnology Development Co (Fuzhou, China). Kallistatin, hyaluronic acid (HA), procollagen III and laminin (LN) ELISA kits were purchased from R&D Systems (Minneapolis, America). Alanine aminotransferase (ALT), aspartate aminotransferaseam (AST), malondialdehyde (MDA) and superoxide dismutase (SOD) kits were purchased from the Biotechnology Research Institute (Nanjing, China). Hydrogen peroxide was purchased from Sigma. Molecular Probe dihydroethidium (DHE) was purchased from Invitrogen (New York, America). Hydrogen peroxide (H_2_O_2_) was prepared as described previously [11].

### Rat models of liver fibrosis and treatment protocol

Male Sprague-Dawley (SD) rats (150–200 g) were obtained from the Experimental Center of Medical Scientific Academy of Fujian. Animal experiments were approved by Animal Ethics committee of Huaqiao University. The animals were cared for in accordance with protocols approved by the Animal Care and Use Committee of Huaqiao University. Animals were housed in a temperature-controlled environment (20–22°C) and 75±2% relatively humidity with a 12 h/12 h light/dark cycle, and acclimatized for one week before the experiment. A total of 40 rats were studied. The rats were divided into five groups, each group included 8 rats. All groups, excluding group 1, received a subcutaneous injection of 10 ml/kg carbon tetrachloride (CCl_4_) dissolved in peanut oil (25%, v/v) twice a week for 7 weeks. Animals in group 1 were subcutaneously injected with the carrier vehicle peanut oil (10 ml/kg daily) only as a negative control and group 2 received CCl_4_ delivered by the vehicle peanut oil as a model control. Group 3, 4 and 5 were injected intraperitoneally with kallistatin, at a dose of 2, 0.5 and 0.125 mg/kg daily, respectively, during the period of CCl_4_ treatment.

At the end of the treatment, all animals were anesthetized with ketamine hydrochloride (30 mg/kg, *iv*) and then sacrificed. Blood samples were immediately collected into tubes and then centrifuged at 3000 g for 10 min at 4°C for serum preparation. Specimens were cut out from the liver and washed immediately with an ice-cold PBS to remove blood. A half of the each specimen was stored at −80°C for future analysis. The other half was fixed in a 10% formalin solution for histopathological analysis.

### Biochemical analysis

The serum levels of AST, ALT and albumin were measured by the commercial colorimetric kits, according to the manufacturer's instructions. Serum hyaluronic acid, type III collagen and LN were determined by ELISA using commercially available kits according to the manufacturer's instructions.

### Hydroxyproline assay

Liver hydroxyproline content was determined by the method reported by Iwaisako *et al* [12]. Hydroxyproline content was expressed as microgram of hydroxyproline per gram liver.

### MDA and SOD assay

The extent of lipid peroxidation was estimated by measurement of MDA formation in the liver homogenates based on the reaction of MDA with thiobarbituric acid (TBA) [13]. Two molecules of chromogenic reagent (2-Thiobarbituric acid) react with one molecule of MDA to yield a stable chromophore. The assay was performed using a MDA kit followed the manufacturer instructions. Briefly, 200 µL TBA and antioxidants reagents from the kits were respectively added into a microcentrifuge tube containing a 100-µL aliquot of each of the liver homogenate and 100 µL of MDA standard solution, and then vortexed. The tubes was incubated at 95°C for 40 min and centrifuged at 4,000×g for 10 min. Then 300 µL of the supernatant from each of the tubes was transferred to a well of a microplate and the absorbance measured at 532 nm using a multi-detection microplate reader (Infinite 200, Tecan). The level of MDA is expressed as nmol MDA/mg homogenate protein. Protein concentration was determined according to Bradford method using bovine serum albumin as a standard.

Liver SOD activity was measured as previously described [14] with slight modification. Briefly, 0.1 mL of the liver homogenate was mixed with 1.5 mL of 20 mM Tris-HCl (containing 1 mM EDTA, pH 8.2), and then 0.1 mL of 15 mM pyrogallol was added. The activity was measured at 420 nm for 3 min and expressed as U/mg protein. One unit was determined as the amount of enzyme that inhibited the oxidation of pyrogallol by 50%.

### Histopathology examinations

Specimens of the liver were fixed in 10% formaldehyde, processed using routine histology procedures, embedded in paraffin, cut in 5 µm sections and mounted on a slide. The samples were stained with hematoxylin and eosin (H&E) and Sirius red respectively. The content of collagen (stained light red on a pale yellow background) was quantified using the histogram module of Photoshop 8.0 software (Adobe).

### Immunohistochemistry

To detect the immunohistochemical localization of α-SMA and TGF-β1, sections from formalin-fixed, paraffin-embedded specimens were deparaffinized and rehydrated in decreasing concentrations of ethylalcohol. All tissue sections were treated with fresh 3% hydrogen peroxide for 20 min, and then washed with PBS. The sections were sequentially incubated with 1% normal blocking serum for 30 min, with rabbit anti-rat α-SMA monoclonal antibody or the rabbit anti-rat TGF-β1 polyclonal antibodies incubate for 60 min, with appropriate HRP-conjugated goat anti-rabbit secondary antibody for 60 min and with DAB as a substrate. All the incubation steps were performed at room temperature and three washes with PBS were applied between the steps. Negative controls were obtained by omitting the primary antibodies. The sections, counterstained with haematoxylin, were then mounted and observed under light microscopy by a blinded pathologist.

### Hepatic stellate cell culture and activation

Hepatic stellate cells (HSCs) were isolated from livers of male SD rats by the discontinuous density gradient centrifugation technique as previously described [11], cultured in DMEM supplemented with 10% fetal bovine serum and 100 U/mL penicillin, 100 µg/mL streptomycin in a dish coated with collagen, and maintained at 37°C in a 5% CO_2_ incubator. The purity of the isolated HSCs was assessed through direct cell counting under a phase-contrast microscope by intrinsic vitamin A autofluorescence and by immunohistochemistry using a monoclonal antibody against desmin. Cell viability was examined by trypan blue dye exclusion. Both cell purity and viability were in higher than 90%.

To characterize the effect of kallistatin on HSCs activation, 1×10^4^ primary HSCs were seeded in a 96-well-plate well with or without kallistatin in culture medium containing 10% FBS and incubated in 37°C. The media were changed every other day. Six days later, activated HSCs were identified with immunocytochemical analysis of rabbit anti-rat α-SMA monoclonal antibody.

### Determination of intracellular Reactive Oxygen Species

Human HSC cell line (LX-2) is a widely used hepatic stellate cell in the fibrosis investigation [15], [16]. The LX-2 cell line was purchased from BioHermes Inc (Jiangsu province, China). LX-2 were cultured in 96-well plates at a density of 1×10^4^ cells/well for 24 h. Kallistatin at different final concentrations (2–50 µg/ml) was added and preincubated for 12 h. Then, the media were replaced with a serum-free medium with 1200 µM H_2_O_2_. Control cells were received a serum-free medium without H_2_O_2_. The cells were incubated for 6 h, and then with addition of 10 µM DHE for 30 min. Intracellular ROS contents were measured by quantifying conversion of DHE to fluorescent ethidium (ETH) [16]. ETH was excited at 535 nm, and emissions were monitored at 635 nm using a photoncounting photomultiplier.

### Statistical analysis

Results were shown as mean±SD. Comparisons among groups were performed by one-way ANOVA, followed by Scheffe's test. The criterion for statistical significance was set at *p*<0.05 and highly significance at *p*<0.01.

## Results

### Kallistatin protected against CCl_4_-induced liver injury

**Figure 1:**
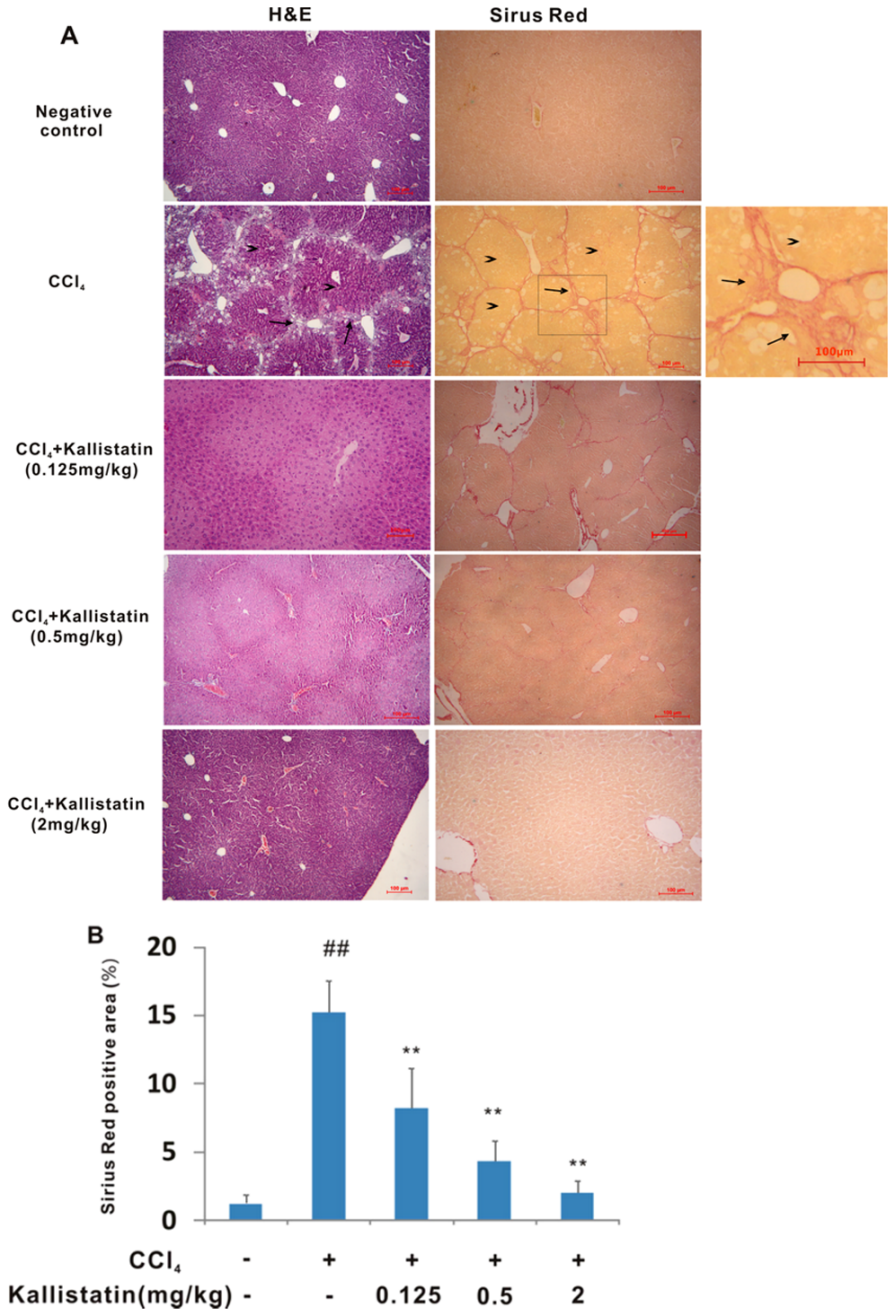
Kallistatin prevents CCl_4_–induced injury to hepatic structure in rats. (A) Representative images of H&E or Sirus red stained sections (original magnifications ×40). Hyperplastic proliferation of hepatocytes in nodular formations (arrowheaded) surrounded by fibrotic septa (arrowed). Scale bars = 100 µm. (B) Collagen deposition was evaluated by Sirius red staining and quantitated by image analysis. Data are expressed as mean±SD (n = 8). ## *p*<0.01 *vs.* negative control; * *p*<0.01 *vs.* model control group.

A rat hepatic fibrosis model with chronic CCl_4_ injection was established, the degree of liver fibrosis was assessed by H&E and Sirius red staining (Figure 1A). Negative control (oil-injected without CCl_4_) rats did not show any liver damage. Following CCl_4_ administration, histological examination of the livers by H&E staining demonstrated a distorted architecture with extensive fibrosis combined with development of micronodules throughout the liver parenchyma. Liver injury was attenuated in the kallistatin-treated groups. Fibrillar collagen deposition as an indicator of liver fibrosis was determined by Sirius red staining. Repeated injection of CCl_4_ induced bridging fibrosis. In contrast, by addition of kallistatin, the pathological progression was attenuated showing fewer and smaller fibrotic nodules. Quantification of Sirius red staining showed obvious increase in collagen accumulation in CCl_4_-induced fibrotic rats compared with the negative control group, while combined administration of kallistatin resulted in a dose-response decrease in staining positive area (Figure 1B). These data supported that kallistatin administration was efficient in reducing pathological collagen deposition and structure damage in the CCl_4_-induced rat hepatic fibrosis model.

**Figure 2:**
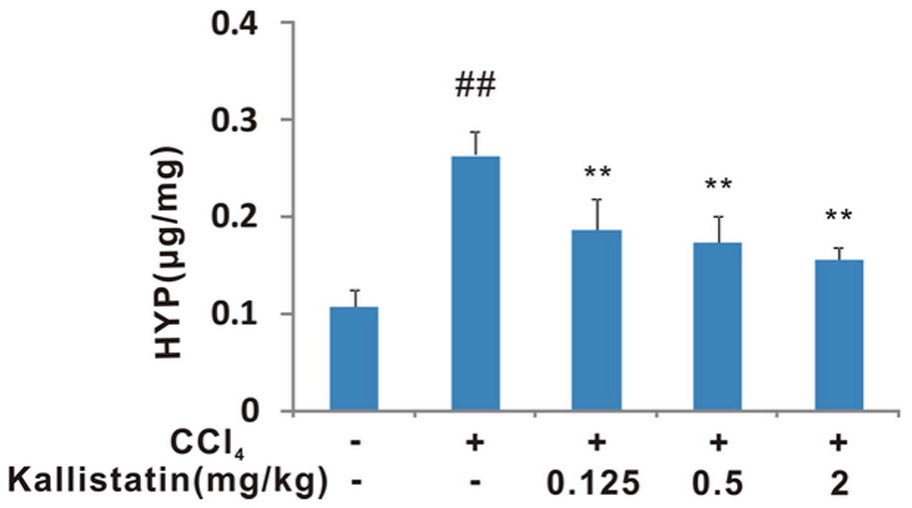
Effects of kallistatin on hepatic hydroxyproline content in CCl_4_-induced liver fibrosis rats in a dose-dependent manner. The results are shown as the mean±SD (n = 8). ## *p*<0.01 vs. negative control; *p<0.05 vs. model control group; ** *p*<0.01 vs. model control group.

The results were further confirmed by analysis of hydroxyproline content in liver. Fibrosis, which is the final result of prolonged liver injury, can be quantified by hydroxyproline analysis and expressed as liver collagen content. Hydroxylation of prolines stabilizes collagen, and increased hydroxyproline content is a marker of fibrosis [17]. The CCl_4_-treated rats had significantly higher hepatic hydroxyproline content than the oil-injected control rats (Figure 2). Whereas, administration of kallistatin reduced the hepatic hydroxyproline content in CCl_4_-induced hepatic fibrosis rats in a dose-dependent manner (Figure 2).

### Kallistatin protected the liver against CCl_4_-induced fibrogenesis

**Figure 3:**
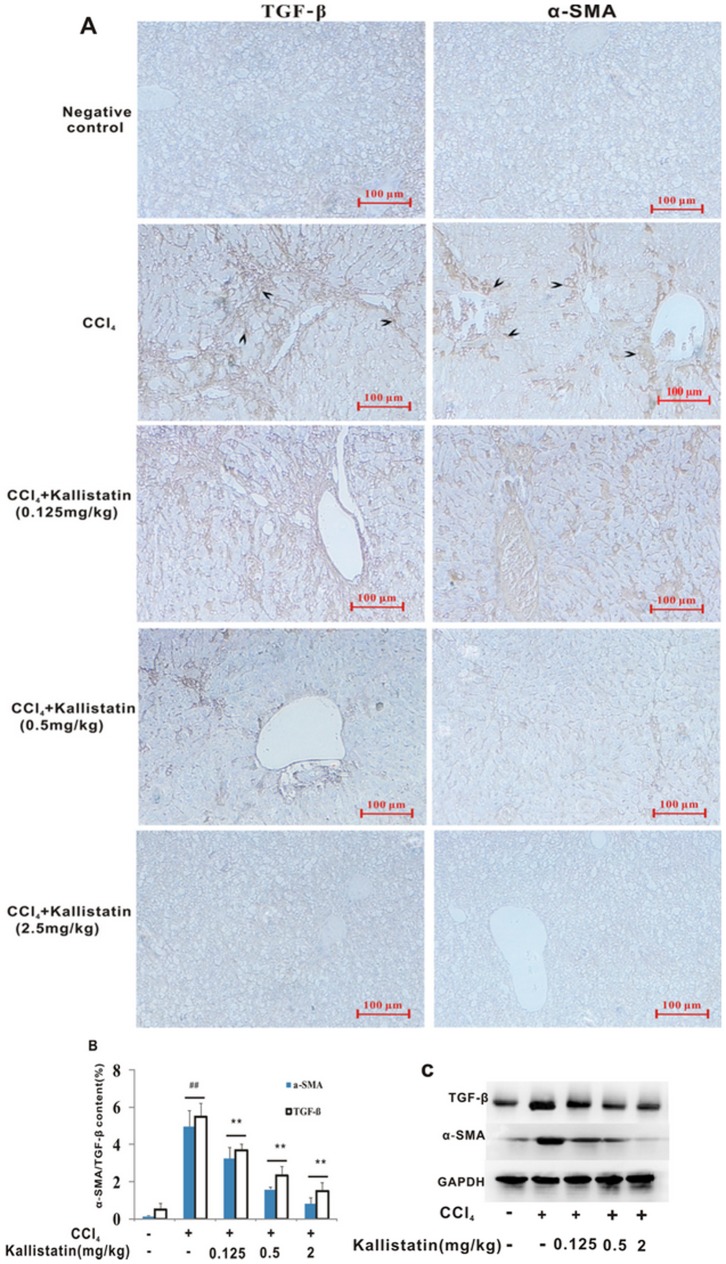
Kallistatin prevents CCl_4_–induced liver fibrogenesis in rats. (A) Representative images of immunohistochemical staining for TGF-β1 (brown in color, arrowheaded) and α-SMA (brown in color, arrowheaded) are shown (original magnifications ×10) respectively. Expression of α-SMA around the periportal fibrotic band areas, central vein and fibrous septa were arrowed. Scale bars = 100 µm. (B) Deposition of α-SMA and TGF- β1 was quantitated by image analysis based on the immunohistochemistry results. Data are expressed as mean±SD (n = 8). ## *p*<0.01 vs. negative control; ** *p*<0.01 vs. model control group. (C) Immunoblotting analysis of α-SMA and TGF- β1 in livers from CCl_4_ alone or plus kallistatin treated rats. Data from immunoblotting were confirmed, showing kallistatin-dependent abrogation of α-SMA and TGF-β1 expression. Immunoblotting and immunohistochemistry results were consistent. Housekeeping proteins GAPDH are useful as loading controls for western blot and protein normalization.

Histological assessment of liver tissue has been the bedrock for diagnosis and staging of fibrosis. However, as a relatively static process of liver injury, fibrosis content alone is not enough to convey the information about the fibrogenic activity. Accordingly, tests that reflect fibrogenesis are important complements to the tests that assess only fibrosis content. As a marker of HSCs activation α-SMA is one of the sensitive indicators of the rate of fibrogenesis [2]. Although several potential non-HSC sources of myofibroblast have been identified, the concept that major myofibroblasts in hepatic fibrosis can be recognized as activated HSCs is still generally accepted [2]. Our results showed that there were hardly any α-SMA positive cells in the negative control (Figure 3). In contrast, considerable expression of α-SMA was detected in the model control group, around the periportal fibrotic band areas, central vein and fibrous septa (Figure 3). Expression of α-SMA in all the CCl_4_ plus kallistatin-treated groups showed a remarkable reduction, compared with the model control group, although higher than that in the negative control.

Pro-inflammatory cytokine TGF-β1 is a central regulator in chronic liver disease, contributing to all stages of disease progression, from initial liver injury through inflammation and fibrosis to cirrhosis and HCC. TGF-β1 is the most potent inducer of collagen I and other matrix constituents, and thus inhibiting its actions remain a major focus of antifibrotic efforts in liver. TGF-β1 expression was significantly increased in the liver sections in the model control group (Figure 3) comparing with the negative control. Expression levels of TGF-β1 in the CCl_4_ plus kallistatin-treated groups were significantly lower than that in the fibrosis model control group, although higher than that in the negative control (Figure 3). These results indicated that kallistatin suppresses the CCl_4_-induced fibrogenic activity related to downregulation of TGF-β1.

### Effect of kallistatin on class I serum biomarkers

**Figure 4:**
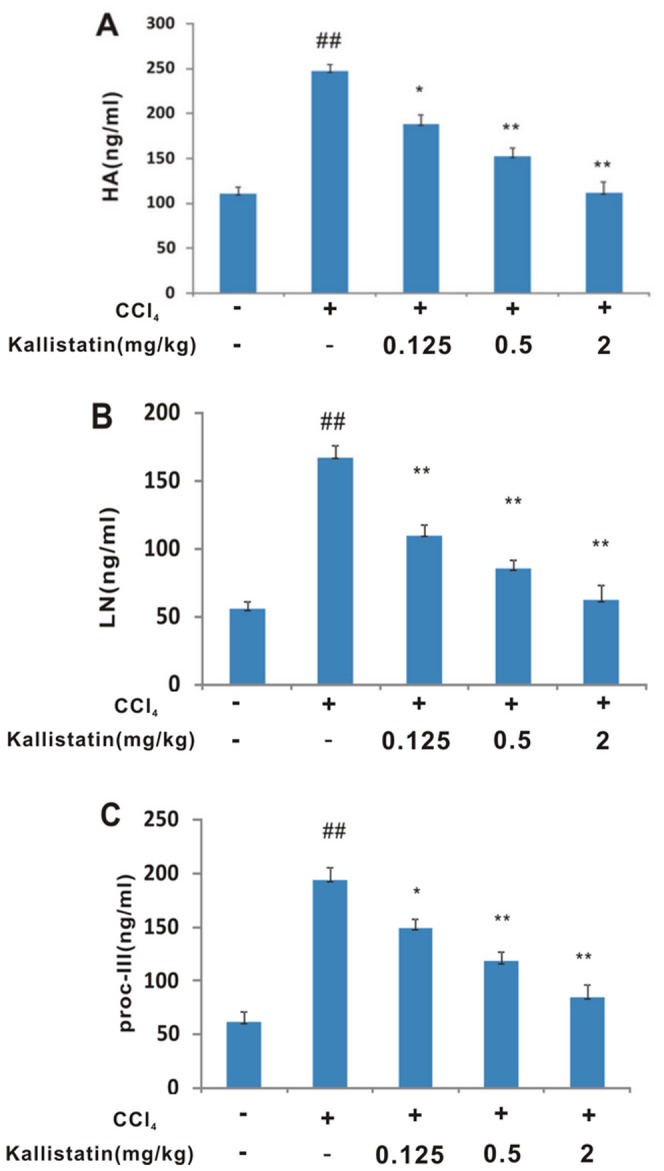
Kallistatin prevents CCl_4_–induced increase of serum HA (A), LN (B) and procollagen III (C) levels in rats. The results are shown as the mean±SD (n = 8). ## *p*<0.01 vs. negative control; *p<0.05 vs. model control group; ** *p*<0.01 vs. model control group.

Fibrosis accumulation is a dynamic process resulting from liver injury. The formation of an interstitial collagen-rich matrix represents a change in both the quality and quantity of the extracellular matrix (ECM). Class I serum biomarkers are associated with the process of fibrogenesis. Their presence in the serum is the result of the turnover of ECM. Several class I markers, such as HA, LN and procollagen III, represent attractive indicators to measure directly the fibrogenic process that leads to clinical complications [15]. CCl_4_ treatment markedly stimulated HA, LN and procollagen III releasing in comparison with the control group (*p*<0.01). The serum HA, LN and procollagen III levels in the three kallistatin groups were significantly lower than that in the model control group (*p*<0.01–0.05) in a dose-dependent manner (Figure 4). These data further demonstrated the efficacy of kallistatin on preventing liver from fibrogenesis.

### Effect of kallistatin on functional reserve of rat liver

Assessment of residual hepatic functional reserve is indispensable for stratifying the severity of liver fibrosis and cirrhosis. No single marker is entirely reliable for predicting residual function, since hepatocytes possess a wide array of different functions. Tests of this type provide more information than simple hepatic histologic features about the progression of liver fibrosis, irrespective of the etiology. Hepatic capacity of protein synthesis is regarded as an important aspect of the hepatic functional reserve [14]. Serum albumin levels are commonly used as an important marker. The administration of CCl_4_ caused severe liver damage characterized by significant decreases (*p*<0.01) in serum albumin, compared with the control group (Table 1). Serum albumin levels in the three kallistatin groups increased significantly, compared to the model control group. There was an obvious dose-respond relationship on the liver function recovery among kallistatin treatment groups.

**Table 1:** Effects of kallistatin on serum AST, ALT and Albumin in CCl_4_-induced liver fibrosis rats (n = 8).

Treatment	AST(U/L)	ALT(U/L)	Albumin(g/L)
negative control	78.99±2.63	66.29±5.29	45.50±3.20
CCl_4_	177.43±4.11##	140.77±15.27##	25.3±2.50##
CCl_4_+kallistatin (0.125 mg/kg)	114.07±5.88**	102.86±17.04**	29.38±2.10*
CCl_4_+kallistatin (0.5 mg/kg)	101.25±4.53**	92.65±10.25**	35.68±4.35**
CCl_4_+kallistatin (2 mg/kg)	85.77±3.51**	81.23±5.99**	42.51±3.090**

The results are showed as mean±SD.

## *p*<0.01 *vs.* negative control;

*p<0.05 *vs.* model control;

**p<0.01 *vs.* model control.

We also measured the serum AST and ALT levels to determine the degree of residual hepatic functional reserve in the rats (Table 1). Consistent with the albumin data, significantly lower levels of the serum AST and ALT were resulted from the addition of kallistatin compared to the fibrosis model group.

### Kallistatin protected from liver oxidative stress

Oxidative stress is known to play a critical role in CCl_4_-induced liver damage and fibrosis formation. MDA is one of the end products from lipid peroxidation, and can be used as a marker of oxidative stress to assess lipid peroxidation. On the other hand, SOD and catalase are important antioxidant enzymes that function as endogenous free radical scavengers. Our data showed that the MDA level was significantly higher in liver homogenates of CCl_4_-intoxicated rats compared with the negative control group (Table 2). Treatment with kallistatin significantly reduced the MDA levels. Conversely, the levers of SOD were significantly increased in the livers of rats in the fibrosis groups treated with kallistatin (Table 2), compared with the fibrosis model group. These data suggest that kallistatin increases SOD activity and suppresses oxidative stress in the liver.

**Table 2:** Effects of kallistatin on MDA and SOD in CCl_4_-induced liver fibrosis rats (n = 8).

Treatment	MDA(nmol/mg)	SOD(nU/mg)
negative control	0.48±0.14	37.13±3.70
CCl_4_	0.95±0.09##	22.66±2.66##
CCl_4_+kallistatin (0.125 mg/kg)	0.72±0.10*	27.45±3.48**
CCl_4_+kallistatin (0.5 mg/kg)	0.59±0.08**	30.56±4.56**
CCl_4_+kallistatin (2 mg/kg)	0.48±0.07**	33.39±3.32**

The results are showed as the mean±SD (n = 8).

## *p*<0.01 *vs.* negative control;

*p<0.05 *vs.* model control group;

**p<0.01 *vs.* model control group.

### Kallistatin scavenged H_2_O_2_ -induced ROS *in vitro*

**Figure 5:**
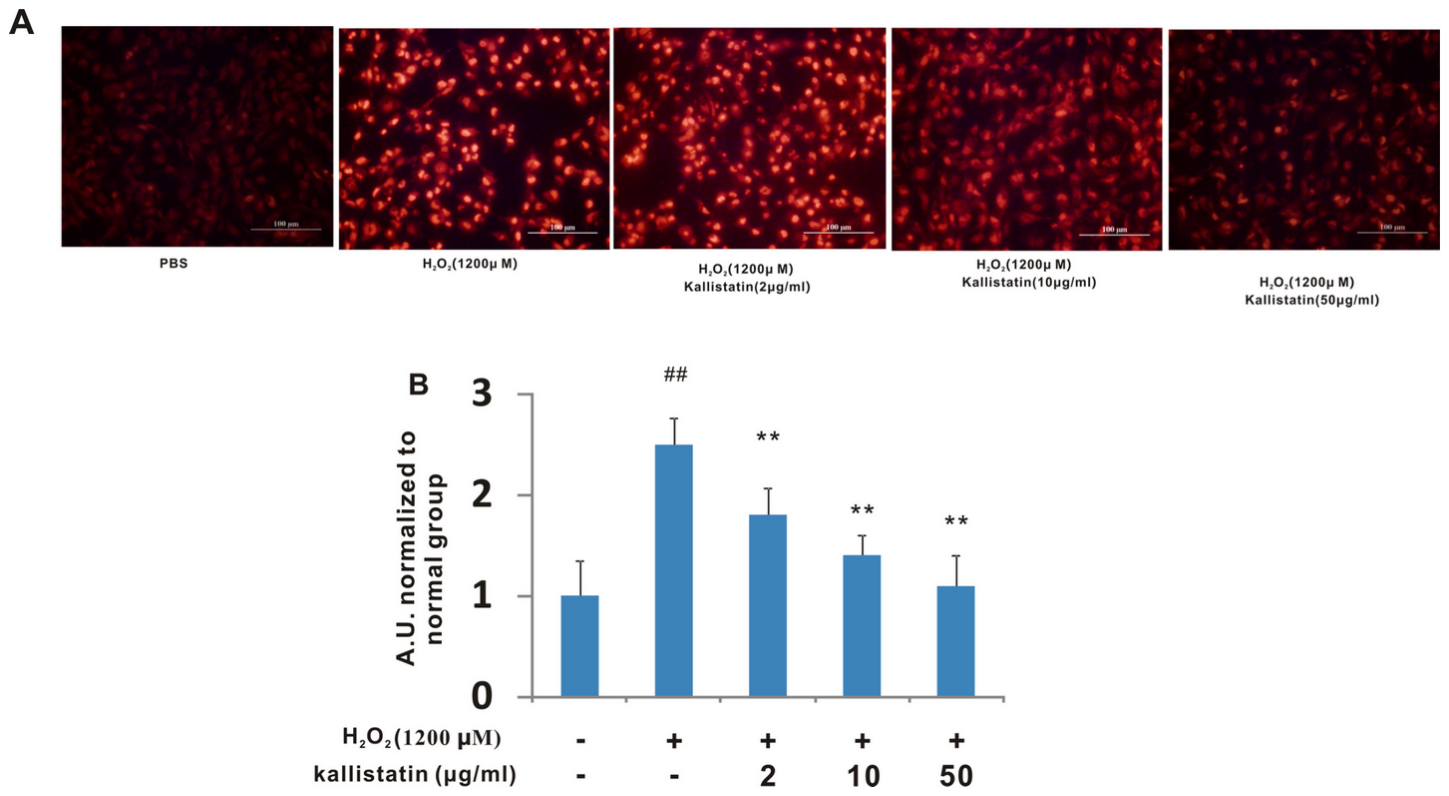
Effect of kallistatin on intracellular ROS levels after H_2_O_2_ exposure in LX-2. Cells were treated without or with kallistatin (2 µg/ml, 10 µg/ml and 50 µg/ml, respectively) prior to H_2_O_2_ challenge. ROS levels were measured using fluorescent probe DCFH-DA from the cells. (A) Representative images of ROS levels. Scale bars = 100 µm. (B) Intensity arbitrary units (A.U.) reflecting the relative value of intracellular ROS levels. Each value represents the mean±SD of triplicates. ## *p*<0.01 vs. normal; **p<0.01 vs. H_2_O_2_ alone.

H_2_O_2_ is widely used to make oxidative stress model *in vitro*. After H_2_O_2_ exposure, oxidative stress results from increased intracellular ROS production and decreased ROS scavenging. To explore the mechanism of the protective effect of kallistatin on oxidative stress-induced damage in LX-2 cells, we examined the effect of kallistatin on intracellular ROS production. To determine whether kallistatin prevents H_2_O_2_-induced ROS generation, the concentration of intracellular ROS was evaluated by the changes in ETH fluorescence intensity. H_2_O_2_ exposure caused an apparent increase in ROS levels compared to control group, whereas pretreatment with kallistatin resulted in reduced ROS levels dose-dependently (Figure 5).

### Kallistatin suppressed the activation of primary cultured HSCs

**Figure 6:**
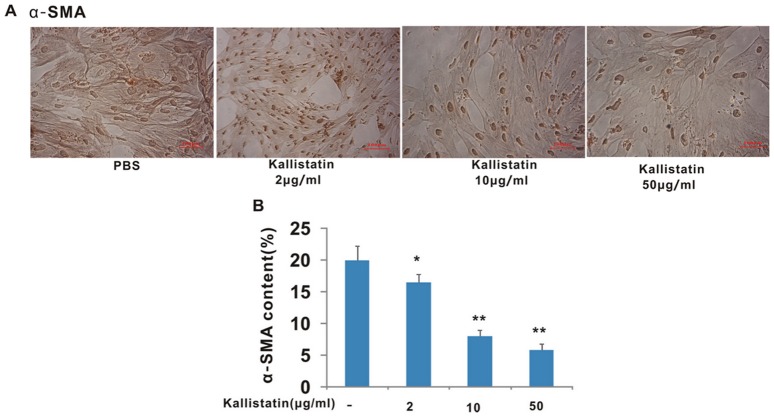
Kallistatin suppressed the expression of α-SMA, the marker of activated HSCs. (**A**) Cultured HSCs on days 6 were fixed in 4% paraformaldehyde and subjected to immunocytochemistry analysis of α-SMA (brown colored). Scale bars = 100 µm. (B) Deposition of α-SMA and TGF- β1 was quantitated by image analysis based on the immunohistochemistry results. Data are expressed as mean±SD (n = 8)*p<0.05 vs. PBS group; ** *p*<0.01 vs. PBS group.

To find whether kallistatin can inhibit HSCs activation, we performed *in vitro* studies in primary cultures of HSCs. Generally, isolated primary HSCs undergo autonomous activation when cultured in plastic dishes, and the activation is associated with the expression of α-SMA. The expression levels of α-SMA were demonstrated with immunocytochemistry analysis. Our data showed that α-SMA was expressed and uniformly distributed in the cytoplasm (Figure 6). The activated HSCs exhibited a stretched morphology with developed stress fibers, which consisted of α-SMA. Kallistatin suppressed α-SMA expression in HSCs dose-dependently.

## Discussion

Chronic liver diseases constitute a global challenge. Current medical treatments for these diseases have achieved limited efficacy. All chronic liver diseases can lead to hepatic fibrosis and eventually liver failure. It is in high demand to find new medicines for the treatment of liver diseases and control of fibrogenesis [18], [19].

Kallistatin is a pleiotropic cytokine which has anti-inflammatory and anti-oxidant properties, and may hold therapeutic promise in prevention of various diseases, including cardiometabolic disorders [20], vascular injury [3], [8], arthritis [5], [21], [22], cancer [5], [23]–[25], kidney fibrosis [4], cardiac hypertrophy and fibrosis [6]. Studies of kallistatin gene delivery have shown significantly alleviated the CCl_4_-induced oxidative stress and inflammatory response, and reduced the liver damage in mouse models [9].

All the *in vivo* experiments with the efficacy of kallistatin on fibrosis were based on kallistatin gene delivery methods [4], [6], [9]. The safety concern on gene delivery vectors slows down the successful translation to the clinic. On the other hand, the recombinant human proteins sector still represents a significant and growing proportion of the overall pharmaceutical market. The recombinant kallistatin contains 100% human sequence. To our knowledge, the present study has for the first time shown the hepatoprotective effect of recombinant kallistatin protein on CCl_4_-induced hepatic fibrosis in rats.

HSCs have been recognized as the main matrix-producing cells in the process of liver fibrosis. The identification of HSCs has been a key development in understanding the process of liver fibrosis. Activation of HSCs is the dominant event in hepatic fibrogenesis, which is characterized by the transformation of quiescent cells into proliferative, fibrogenic, and contractile myofibroblasts. The expression level of α-SMA is a sensitive indicator of the fibrogenic myofibroblasts. We have found that the recombinant human kallistatin effectively inhibited the myofibroblast-like activation of isolated rat HSCs and reduced the expression of α-SMA in vitro. We have tested the hypothesis that the suppression of myofibroblast-like transformation and activation of HSCs upon kallistatin treatment may help alleviate CCl_4_-induced liver fibrosis *in vivo*. Indeed, the *in vivo* data showed that CCl_4_-induced liver injury and collagen deposition in the experimental groups were accompanied by high expressions of TGF-β1, indicating that the TGF-β1 active HSCs classic pathway is active. Damaged resident liver cells triggered a profibrogenic response that stimulates TGF-β1 expression, and converts latent TGF-β1 into the active form, which provokes early HSCs activation. The recombinant human kallistatin was administered at the initial stage of hepatic fibrosis, and continued on a period of 7-week, so that an adequate biological effect could be exhibited. The recombinant human kallistatin treatment significantly reduced the expression levels of TGF-β1 and α-SMA in liver along with the progress of fibrogenesis in the rat model, indicating that the recombinant kallistatin had an inhibitory effect on hepatic fibrosis (Figure 1), at least partly via down regulation of the expressions of TGF-β1 and suppression of HSCs activation (Figure 3).

Since persistent inflammation and oxidative stress always precede and accompany with liver fibrosis, drugs that inhibit the inflammatory cascade and ROS generation typically have antifibrotic effects. Recent findings have indicated that kallistatin may play an important role in the protection against oxidative stress-induced inflammatory responses and organ damage. In a highly reproducible rat resistant hepatocyte model, kallistatin was not detectable or at very low levels in nodule tissue compared with normal liver by proteomics study [26]. A significantly reduced kallistatin level was seen in plasma samples from patients with chronic, severe liver disease [27], suggesting that kallistatin is produced mostly in the liver and can be consumed during inflammation in the liver. The results of the present study demonstrated that cells pretreated with kallistatin decreased the formation of ROS products after exposure to H_2_O_2_ (Figure 5), which supported the idea that kallistatin protected cells from oxidative stress-induced cytotoxicity by its antioxidant property. SOD is considered to be the most relevant enzyme involved in detoxification of H_2_O_2_ and protection of hepatocytes from oxidative stress. The inhibited activities of SOD induced by CCl_4_ *in vivo* were attenuated by kallistatin. The results obtained here provide strong evidence that kallistatin attenuated ROS formation, and exerted a protective effect against CCl_4_-induced liver injury.

Chronic liver disease results in hepatic fibrosis and cirrhosis. Complications of cirrhosis include ascites, portal hypertention, encephalopathy, liver failure, and high risk of HCC. Despite no drug has yet clinically available as an effective antifibrotic agent, the progress in the research on cellular and molecular biology of the liver fibrosis and cirrhosis has provided an opportunity to develop antifibrotic therapies. The findings reported here suggest kallistatin is effective to reduce inflammation and immune response, and to attenuate ROS induced by CCl_4_ injection. Further investigation toward treating patients with chronic liver diseases should be promoted.
